# Exercise dose, measurement reliability, and mechanistic inference in fibromyalgia trials: a critical appraisal

**DOI:** 10.1016/j.lana.2026.101384

**Published:** 2026-02-09

**Authors:** André Pontes-Silva

**Affiliations:** Federal University of Sao Carlos (UFSCar), São Carlos, SP, Brazil

Fregni et al.^1^ published a trial examining effects of aerobic vs. non-aerobic exercise combined with tDCS on CPM and clinical outcomes in fibromyalgia. Authors address mechanistic/clinical endpoints within a rigorous framework.^1^ However, there are interrelated methodological/conceptual concerns that substantially affect the study's internal validity, theoretical coherence, and translational interpretation.

## Conceptual alignment among hypotheses, outcomes, and interpretation

Study^1^ is framed around a clear a priori hypothesis: that aerobic exercise combined with active tDCS would outperform non-aerobic exercise with sham stimulation in both CPM and clinical outcomes. However, the results show that (i) tDCS selectively improved CPM, (ii) exercise—regardless of intensity—improved clinical symptoms, (iii) CPM changes did not mediate clinical improvements, and (iv) aerobic exercise was not clinically superior to non-aerobic exercise.

This pattern shows that the integrated hypothesis is unsupported, yet this dissociation is not treated as theoretically meaningful. The study^1^ would be strengthened if the authors reframed their aims around three questions: (i) which interventions modulate descending inhibitory mechanisms, (ii) which improve clinical symptoms, and (iii) whether these processes are necessarily coupled.^1^

Treating this dissociation as central would strengthen the manuscript's theoretical depth. This aligns with contemporary biopsychosocial models of pain, which challenge linear assumptions between biomarkers and subjective experience.^1^

## Exercise dose: absence of total work quantification and equalization

A central study's conclusion is that aerobic and non-aerobic exercise produced comparable clinical improvements. However, the authors neither reported nor controlled total work, cumulative mechanical load, or energy expenditure between groups.^1^

Physiologically, exercises cannot be meaningfully compared without quantifying cumulative dose.^2^ Time-matched protocols are not dose-matched. A 30-min session at 60–70% HRmax imposes a fundamentally different stimulus than one at <40% HRmax.^1^^,^^2^

Because many adaptations depend primarily on cumulative workload rather than nominal modality, the absence of dose quantification weakens causal inference. Without this information, it is unclear whether the apparent equivalence reflects a modality-independent effect or an unmeasured dose convergence.^2^

Thus, the design supports not the conclusion that “exercise modality does not matter,” but rather that two protocols, matched for time and supervision—but not for physiological dose—produced similar outcomes.^2^

## CPM as a primary mechanistic outcome: absence of reliability metrics

A second major issue concerns CPM as the primary mechanistic endpoint. CPM is a psychophysical construct that relies on multiple procedural components—including individualized calibration, standardized instructions, precise timing, and subjective ratings—and is therefore highly sensitive to assessor-dependent variability.^3^

Yet, the study^1^ reports neither inter- nor intra-rater reliability, nor clarifies whether a single blinded assessor conducted all measurements. This omission affects internal validity. When the reliability of a primary mechanistic outcome is unknown, part of the observed variance may reflect procedural inconsistency rather than true biological change.^3^

This is relevant because the authors conclude that CPM changes did not mediate clinical improvement. Without reliability metrics, it is unclear whether this dissociation reflects genuine neurophysiological uncoupling or differential measurement precision across outcomes.^1^^,^^3^

## Interpretation: reframing limitations as theoretical contributions

The study's most innovative finding is not that tDCS improves CPM or that exercise improves symptoms per se,^1^ but that enhanced descending inhibitory capacity does not necessarily translate into short-term clinical improvement in fibromyalgia.^4^

This challenges linear biomarker-to-symptom models and aligns with contemporary views on subjective experience. However, this dissociation is currently framed as a disappointing or null result rather than as a central theoretical contribution.^4^

Likewise, the finding that non-aerobic exercise yields comparable clinical benefits reflects not a failure of aerobic superiority, but evidence that low-intensity, accessible, and more tolerable activity may be clinically meaningful—an insight with direct implications for real-world care.^1^^,^^4^

## Methodological transparency and external validity

Additional aspects warrant explicit discussion. First, the “non-aerobic” condition functions as a low-intensity active control rather than a placebo.^5^^,^^6^ Framing this as a strength—rather than an implicit limitation—would improve interpretability.^5^^,^^6^

Second, the sample is predominantly female, predominantly White, and drawn from a single center. A brief, explicit discussion of generalizability and the need for replication in more diverse populations would strengthen the study's external validity.^1^

Third, the follow-up window is relatively short. Because neuroplastic and clinical processes unfold on different time scales, mechanistic changes may precede or outlast symptoms. This potential temporal decoupling warrants explicit consideration.^1^

Finally, future reports could benefit from more granular data visualization (e.g., individual CPM trajectories, ΔCPM–Δpain scatterplots) and from reporting minimal clinically important differences for key outcomes (e.g., FIQR, BPI, PROMIS).^7^^,^^8^

## Contributors

Pontes-Silva A—Conceptualization, Formal Analysis, Investigation, Methodology, Validation, Visualization, Writing (original draft, review, and editing).

## Declaration of interests

Prof. André Pontes-Silva, PhD, serves as a reviewer for the *Lancet Group*. The author declares no further competing interests.
